# Incidence and Risk Factors of Postoperative Pulmonary Complications in Noncardiac Chinese Patients: A Multicenter Observational Study in University Hospitals

**DOI:** 10.1155/2015/265165

**Published:** 2015-03-02

**Authors:** Yue Jin, Guohao Xie, Haihong Wang, Lielie Jin, Jun Li, Baoli Cheng, Kai Zhang, Andreas Hoeft, Xiangming Fang

**Affiliations:** ^1^Department of Anesthesiology, The First Affiliated Hospital, School of Medicine, Zhejiang University, Hangzhou 310003, China; ^2^Intensive Care Unit, The Sir Run Run Shaw Hospital, School of Medicine, Zhejiang University, Hangzhou 310016, China; ^3^Department of Anesthesiology, The First Affiliated Hospital, Wenzhou Medical College, Wenzhou 325000, China; ^4^Department of Anesthesiology, The Second Affiliated Hospital, Wenzhou Medical College, Wenzhou 325027, China; ^5^Department of Anaesthesiology and Intensive Care Medicine, University of Bonn, 53127 Bonn, Germany

## Abstract

*Purpose*. To assess the incidence of postoperative pulmonary complications (PPCs) in Chinese inpatients, and to develop a brief predictive risk index. *Methods*. Between August 6, 2012, and August 12, 2012, patients undergoing noncardiac operations in four university hospitals were enrolled. The cohort was divided into two subsamples, cohort 1 to develop a predictive risk index of PPCs and cohort 2 to validate it. *Results*. 1673 patients were enrolled. PPCs were recorded for 163 patients (9.7%), of whom the hospital length of stay (LOS) was longer (*P* < 0.001). The mortality was 1.84% in patients with PPCs and 0.07% in those without. Logistic Regression modeling in cohort 1 identified nine independent risk factors, including smoking, respiratory infection in the last month, preoperative antibiotic use, preoperative saturation of peripheral oxygen, surgery site, blood lost, postoperative blood glucose, albumin, and ventilation. The model was validated within cohort 2 with an area under the receiver operating characteristic curve of 0.90 (95% CI 0.86 to 0.94). *Conclusions*. PPCs are common in noncardiac surgical patients and are associated with prolonged LOS in China. The current study developed a risk index, which can be used to assess individual risk of PPCs and guide individualized perioperative respiratory care.

## 1. Introduction

More than 230 million major surgical procedures are undertaken each year worldwide [1] and postoperative complications imposed a significant clinical and economic burden to surgical patients as well as the public health systems [2, 3]. Postoperative pulmonary complications (PPCs) are common postoperative complications that occur in 2% to 40% of patients and are associated with increased morbidity, mortality, and length of stay (LOS) [3–9]. In noncardiac patients, PPCs occur more frequently than cardiac complications [10]. Though it came to wide attention in recent years, the literature investigating the incidence and outcome of PPCs in Chinese inpatients remains scarce.

It is known that PPCs have a multifactorial etiology and had been defined broadly, including respiratory tract infection, pneumonia, respiratory failure, atelectasis, pleural effusion, pneumothorax, bronchospasm, and aspiration pneumonitis [11]. Previous studies demonstrated that PPCs were associated with a series of perioperative risk factors, such as age, smoking, chronic obstructive pulmonary disease (COPD), type of surgery, and serum albumin [4, 6, 7, 11–14]. A majority of these risk factors can be intervened and improved [15–17]. Therefore, identifying perioperative risk factors of PPCs is an important step toward improving quality of care in surgical patients, which has been already explored in several studies [11, 12, 18].

In the United States, Arozullah et al. developed a multifactorial risk index to predict the postoperative pneumonia after major noncardiac surgery [12]. In Canada, McAlister et al. paid attention to the nonthoracic surgery [7]. They found the incidence of PPCs is 8% and identified the preoperative risk factors. Dupont et al. also investigated five independent predictive factors of postoperative pneumonia in France [19]. A recently published risk-prediction equation for PPCs was a significant advance in this field, which identified seven independent risk factors for PPCs by Canet et al. [11]. The researchers further tested this predictive risk score in a large European cohort and found this risk score performed differently between geographic areas [20]. In China, the most populous developing country in the world, however, very limited information about PPCs has been reported so far. Since China is different from the USA and European countries in geographic areas, race, disease spectrum, and social psychological background, investigations assessing the incidence and characteristics of PPCs in Chinese surgical cohorts are indispensable. Hence we conducted the present study to assess the incidence and risk factors of PPCs after noncardiac surgery in China and to develop a risk index of PPCs which is applicable for Chinese inpatients. The results of the present study would help health care providers to understand the existing situation of PPCs in China, to identify high-risk patients from the generic surgical population, and to guide individualized perioperative respiratory care.

## 2. Materials and Methods

### 2.1. Study Settings and Patients

We conducted a prospective, multicenter, and observational study of inpatients undergoing noncardiac surgical procedures. This study was performed at 4 university hospitals located in Zhejiang province, China (the First Affiliated Hospital, Zhejiang University School of Medicine, the Sir Run Run Shaw Hospital, Zhejiang University School of Medicine, the First Affiliated Hospital, Wenzhou Medical College, and the Second Affiliated Hospital, Wenzhou Medical College). The inpatients undergoing surgical procedures between August 6, 2012, and August 12, 2012 were enrolled into the study. All patients would receive routine care, and no research-related intervention would be introduced.

### 2.2. Inclusion and Exclusion Criteria

All noncardiac operations performed under general, spinal, epidural, or regional anesthesia were eligible for inclusion. The exclusion criteria were as follows: (1) younger than 18 years; (2) pregnancy; (3) organ transplantation; (4) procedures performed under local nerve anesthesia; (5) procedures outside the operating room; (6) outpatient procedures (who had an LOS in hospital less than 24 hours); (7) reoperation related to a previous surgical complication; (8) patients with preoperatively intubated trachea.

### 2.3. Data Collection

Two trained anesthesiologists were assigned at each center to collect the following data: (1) generic information: date of surgery and hospital admission/discharge, age, gender, American society of anesthesiologists (ASA) physical status, height and weight, smoking status, alcohol use, and chronic comorbid disease; (2) preoperative variables: respiratory infection in the last month, antibiotic use, nasogastric tube, saturation of peripheral oxygen (SpO_2_), and laboratory results (leucocytes count, neutrophil, hemoglobin, serum creatinine, serum albumin, and fasting blood glucose); (3) intraoperative variables: anaesthetic technique, surgery (type, site, and duration), nasogastric tube, bladder catheter, central venous catheter, blood loss, blood transfusion, pulmonary, and cardiovascular complications; (4) postoperative variables: clinic (SpO_2_, mechanical ventilation, vasoactive drugs) and laboratory results within 2 hours after surgery. After operation, surgeon and nurses visited the patients every day and recorded incidence of PPCs. The PPCs were defined in Table 1 [11]. Postoperative mortality was defined as death within 60 days of surgery.

### 2.4. Outcomes

The primary outcome was the occurrence of PPCs, the postoperative LOS, and the postoperative mortality rate. The secondary outcome is the predictive risk index of PPCs.

### 2.5. Statistical Analysis

Quantitative data were presented as means and standard deviations (SD) or median and interquartile range (IQR; from 25th to 75th percentiles) as appropriate. Qualitative data were reported as *N* (%). Student's *t*-test or Mann-Whitney *U* test were used for comparison of continuous variables and the chi-squared test or Fisher's exact test for categorical variables to test the relationship between each potential risk factors and PPCs.

In order to develop a risk index of PPCs, enrolled patients were further divided into two cohorts. Cohort 1 composed of patients from two hospitals and was used to develop the predictive index, while cohort 2 was used to validate the index. In cohort 1, risk factors were firstly analyzed using univariate analysis (*P* < 0.05). Then a multivariate logistic regression was conducted in cohort 1 with the occurrence of PPCs as the dependent factor, incorporating all risk factors on the basis of correlation coefficients between variables lower than 0.4. The forward LR mode was adopted in the process of regression. The adjusted odds ratios (OR) and the confidence intervals (CI) were also calculated. A brief predictive index was then calculated by multiplying the regression (*β*) by 10 and rounding off to the nearest integer [12]. The brief predictive index then validated in cohort 2 to evaluate the model's discriminatory capability, and the area under the receiver operating characteristic (ROC) curve was displayed (*c*-statistic).

Data were analyzed using SPSS 16.0 (SPSS inc., Chicago, USA). All these tests were two-tailed and statistical significance was considered when a *P* value was less than 0.05.

## 3. Results

### 3.1. Demographic and Clinical Characteristics

**Figure 1:**
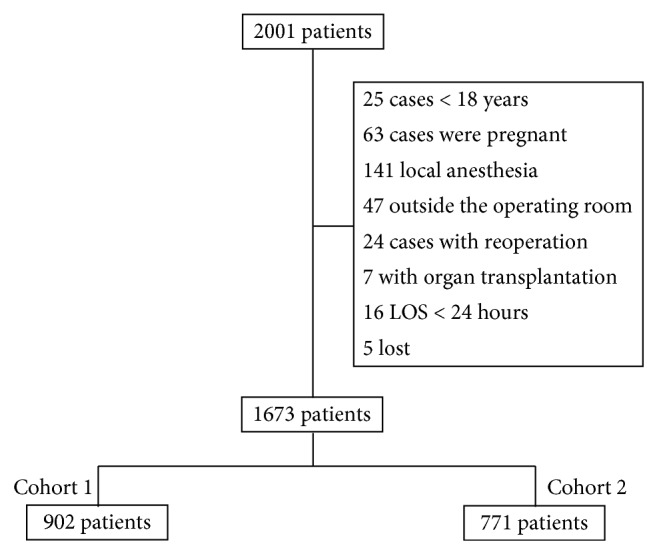
Flow-chart of study population. LOS: the length of stay.

Between August 6, 2012, and August 12, 2012, 2001 patients were undergoing noncardiac surgery, of which 328 were excluded according to the inclusion criteria or lost. Consequently, the final sample included in the statistical analysis consisted of 1673 patients, 902 in cohort 1 and 771 in cohort 2 (Figure 1). The basic characteristics of the study subjects are detailed in Table 2.

### 3.2. PPCs, LOS, and Mortality

A total of 163 (9.7% of the 1673 patients) patients were recorded with 237 episodes of PPCs. Postoperative respiratory failure developed in 30 patients (1.8%), bronchospasm in 48 (2.9%), pleural effusion in 7 (0.4%), respiratory infection in 131 (7.8%), atelectasis in 19 (1.1%), cardiopulmonary edema 1 (0.06%), and pneumothorax in 1 (0.06%). Most PPCs occurred after upper gastrointestinal surgery (40.4%), followed by thoracic (38.8%), neurosurgery (22.2%), kidney (14.3%), hepatobiliary (13.5%), and lower gastrointestinal surgeries (11.8%). The median postoperative LOS was longer in patients with PPCs (16 days, IQR 10–23 days) than in those without PPCs (8 days, IQR 5–13 days) (*P* < 0.001), especially in orthopaedics (*P* = 0.03), breast (*P* < 0.001), gynaecology (*P* < 0.001), lower gastrointestinal (*P* < 0.001), and hepatobiliary (*P* < 0.001) surgery. The detailed information on the characteristics of PPCs and LOS was shown in Table 3.

Four (0.24%) patients died in the hospital, 3 (1.84%) of the 163 patients with PPCs and 1 (0.07%) of the 1510 patients without PPCs; the mortality was significantly higher in patients with PPCs than those without (*P* < 0.001).

### 3.3. Risk Factors and PPCs

**Figure 2:**
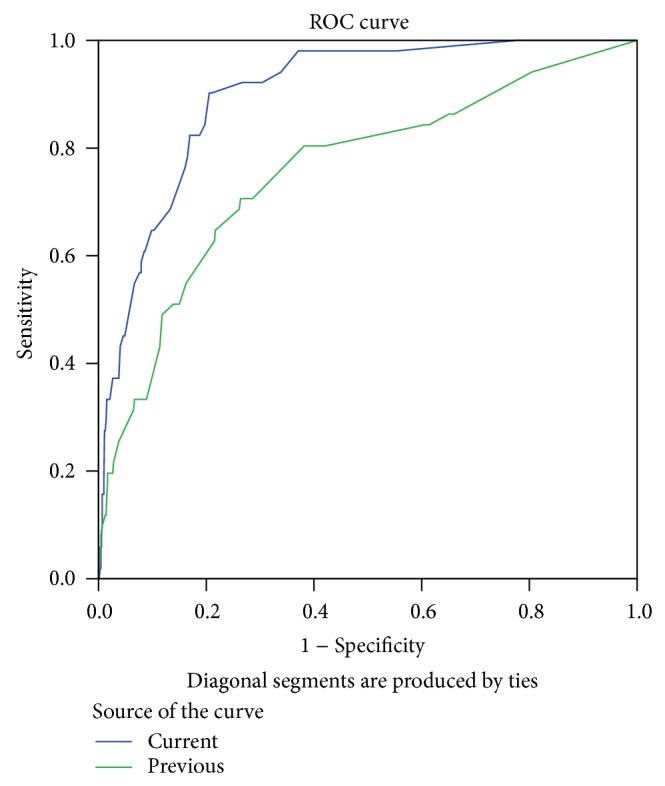
Receiver operating characteristic (ROC) curves drawn for the current model and previous index (Canet's) in cohort 2. *c*-statistic for the area under the ROC curve (AUC) = 0.90 (95% CI, 0.86 to 0.94) drawn from the current index and AUC = 0.76 (95% CI, from 0.68 to 0.83) drawn from the previous index.

The variables having a statistically significant impact on the incidence of PPCs detected from the cohort 1 are shown in Table 4. Then the independent variables were entered into the logistic regression model, except for these have high collinearity with others (intraoperative blood loss and duration of surgery; intraoperative blood loss and central venous catheter; intraoperative blood loss and blood transfusion; postoperative ventilation and vasoactive drug; pre- and intraoperative nasogastric tube; pre- and postoperative hemoglobin). Multivariable logistic regression indicated that 9 of those potential predictors were present in the final model. The raw and adjusted odds ratios for the nine variables are shown in Table 5, which also shows the brief predictive index derived from the *β* coefficient for each variable. This nine-variable regression model had good discrimination and calibration values in cohort 1 (*c*-statistic 0.91, 95% CI, and from 0.89 to 0.94). The ROC curves and the *c*-statistics for the validation subsamples (cohort 2) are shown in Figure 2. The brief predictive index developed in the present study has potential advantage in predicting PPCs in Chinese inpatients (better *c*-statistic than the risk index in the study of Canet et al.) [11]. The most relevant cut point was the score of 13 (sensitivity 90.2%, specificity 79.4%).  Table 6 shows the incidence of PPCs by risk index score, the number of patients in each risk class, and the actual incidence of PPCs in cohort 1 and cohort 2.

### 3.4. Strategies to Reduce PPCs

Opportunities to reduce risk for PPCs occur throughout the perioperative period.  Table 7 summarizes perioperative interventions that have been recommended to decrease the risk of PPCs [15, 16, 21, 22].

## 4. Discussion

This prospective study assessed the 9.7% incidence of PPCs after noncardiac surgery in Chinese university hospitals and found increased hospital LOS by 8 days in patients with PPCs than those without. Furthermore, we identified the perioperative risk factors of PPCs and developed a brief risk index for predicting PPCs in Chinese inpatients.

In the current study, the incidence of PPCs after noncardiac surgery and the hospital LOS in patients with PPCs were comparable to the incidence observed in some previous studies [11, 23–25]. The high incidence of PPCs and the increased hospital LOS indicating PPCs is also an important public health issue demanding nationwide attention in China. Unexpectedly, we found the postoperative mortality in patients with PPCs was lower than several previous results [3, 11]. This may be in association with the higher percentage of ASA class 1 to 2 patients and younger median age. Firstly, the medical resources in China were mainly concentrated in larger hospitals, especially the university hospitals and tertiary hospitals. So the patients always chose to go to large hospitals directly, even if they only have a cold or want to remove a small lipoma on body surface. Secondly, the percentage of emergency surgery in the present study was low, since none of the four studied hospitals had a major trauma center.

Nine independent risk factors were finally selected to participate in the brief predictive index for PPCs, including smoking, respiratory infection in the last month, antibiotic use, SpO_2_, surgery site and blood lost, blood glucose, albumin, and ventilation.

Among the preoperative risk factors, smoking, a history of respiratory infection in the last month, SpO_2_, and antibiotic use are strong PPC risk factors. Smoking and respiratory infection may lead to local changes in airway reactivity, pulmonary function, and residual impairment of immunity, which could increase the risk of PPCs for several folds [6, 7]. SpO_2_ is an easily recorded objective measure, which reflects the respiratory function as well as cardiovascular functional status [11]. Antibiotic use is a protective factor in the current cohort. It seems to be helpful to use prophylactic antibiotic, especially in these high-risk patients.

The intraoperative risk factors were identified in the present study including surgery site and blood loss, which were similar to many previous studies [4, 7, 14, 26]. Thoracic and abdominal surgeries are always performed via a large painful incision associated with obviously changes in lung compliance and functional residual capacity. As a result, the incidence of PPCs in these surgeries is much higher than others. Significant intraoperative blood loss gives rise to haemodynamic instability and relative ischaemia and so causes ischaemia-reperfusion injury, which can lead to organ dysfunction.

Factors in early stage of postoperative may have a better performance in predicting PPCs. We found postoperative mechanical ventilation, albumin, and blood glucose could increase the risk of PPCs. Among them, ventilation is the most important risk factor for PPCs. Researchers found an increased risk of respiratory events in patients with ventilation [26, 27]. It is important to follow the guidelines for the management of ventilation and the prevention of nosocomial infection.

A brief risk score based on these factors was calculated to predict the incidence of PPCs. In Spain, Canet and coworkers also developed a similar risk index of PPCs [11]. Three of these risk factors (preoperative SpO_2_, respiratory infection in the last month, and surgery site) were also identified in the current study. What is more, the brief predictive index developed in the present study has potential advantage in predicting PPCs in Chinese inpatients (better *c*-statistic than the risk index of Canet). This disparity may be due to the different geographic areas, ethnic, cultural, socioeconomic, and political differences between China and Spain. In fact, the risk index developed by Canet also performed differently between the Western Europe sample and the Eastern Europe sample [20]. In general, the current brief predictive index predicted the risk of PPCs well in both the development and validation cohorts. These findings suggest patients with high risk of PPCs should be closely monitored and early intervened if the risks factors could be modified.

Several limitations in our study should be acknowledged. First of all, only four university hospitals participated. This may result in some bias. Further studies targeting general Chinese hospitals with a larger population are still needed. Another limitation is that the 7-day study period was arbitrarily decided. All patients were enrolled in August. Englesbe et al. demonstrated a significant seasonal variation in surgical morbidity and mortality [28]. They found a dramatic worsening of surgical mortality in July, which was attributed to the influx of inexperienced trainees. However, Ehlert and colleagues refuted the “July Phenomenon” in a larger population [29]. In most hospitals in China, July is marked by an influx of intern. Instead of managing patients directly, they only do the paperwork. Therefore, they may have little effect on the present results. Further study is needed to confirm the impact of seasonal variation in surgical morbidity and mortality.

## 5. Conclusion

The present prospective, multicenter study found there was high incidence of PPCs which increased hospital LOS in noncardiac surgical inpatients in China. We identified nine objective and easily assessed factors associated with the occurrence of PPCs. A simple risk index based on these factors predicted the development of PPCs. This brief predictive index may be useful for clinicians in estimating patients' risk for PPCs and guide individualized perioperative respiratory care.

## Figures and Tables

**Table 1:** Definitions of postoperative pulmonary complications.

*Respiratory infection *	
Treatment with antibiotics for a respiratory infection, plus at least one of the following criteria: new or changed sputum, new or changed lung opacities, fever, and leukocyte count >12,000/mm^3^	

*Respiratory failure *	
Postoperative PaO_2_ <60 mmHg on room air, a ratio of PaO_2_ to inspired oxygen fraction <300, or SaO_2_ <90% and requiring oxygen therapy	

*Pleural effusion *	
Chest radiograph demonstrating blunting of the costophrenic angle, evidence of displacement of adjacent anatomical structures, or (in supine position) a hazy opacity in one hemithorax with preserved vascular shadows	

*Atelectasis *	
Collapse of the alveoli, lung opacification with shift of the mediastinum, hilum, or hemidiaphragm toward the affected area, and compensatory overinflation in the adjacent nonatelectatic lung	

*Pneumothorax *	
A collection of air in the pleural space (the area with no vascular bed surrounding the visceral pleura)	

*Bronchospasm *	
Newly detected expiratory wheezing treated with bronchodilators	

*Aspiration pneumonitis *	
Acute lung injury after the inhalation of regurgitated gastric contents	

PaO_2_: partial pressure of oxygen in arterial blood; SaO_2_: arterial oxyhemoglobin saturation.

**Table 2:** Demographic and clinical characteristics.

	Overall	Cohort 1	Cohort 2	*P* ^*^
	(*n* = 1673)	(*n* = 902)	(*n* = 771)	
Male, *n* (%)	770 (46.0)	436 (48.3)	334 (43.3)	0.04
Age, yr	49 (37–60)	48 (36–60)	49 (39–60)	0.69
Education, yr	9 (6–9)	9 (6–9)	9 (9–14.5)	0.31
Smoking status, *n* (%)				0.85
Never smoker	1397 (83.5)	753 (83.5)	644 (83.5)	
Former smoker	222 (13.3)	118 (13.1)	104 (13.5)	
Current smoker	54 (3.2)	31 (3.4)	23 (3.0)	
Drinker, *n* (%)				0.47
Never drinker	1487 (88.9)	804 (89.1)	683 (88.6)	
Former drinker	152 (9.1)	83 (9.2)	69 (8.9)	
Current drinker	34 (2.0)	15 (1.7)	19 (2.5)	
Body mass index, kg/m^2^	22.3 (20.3–24.3)	22.1 (20.3–24. 2)	22.4 (20.3–24.3)	0.85
Preoperative SpO_2_, %	98 (98-99)	98 (98-99)	98 (98-98)	0.45
Respiratory infection in the last month, *n* (%)	28 (1.7)	16 (1.8)	12 (1.6)	0.85
ASA physical status, *n* (%)				0.43
1	830 (49.6)	464 (51.4)	366 (47.5)	
2	722 (43.2)	380 (42.1)	342 (44.4)	
3	114 (6.8)	54 (6.0)	60 (7.8)	
4	5 (0.3)	3 (0.3)	2 (0.3)	
5	2 (0.1)	1 (0.1)	1 (0.1)	
Emergency surgery, *n* (%)	102 (6.1)	55 (6.1)	47 (6.1)	0.99
Anesthesia, *n* (%)				0.24
General and combined^#^	1251 (74.8)	664 (73.6)	587 (76.1)	
Neuraxial/regional	422 (25.2)	238 (26.4)	184 (23.9)	
Surgical site, *n* (%)				<0.001
Peripheral	1015 (60.7)	620 (68.7)	395 (51.2)	
Abdominal	600 (35.9)	243 (26.9)	357 (46.3)	
Intrathoracic	58 (3.5)	39 (4.3)	19 (2.5)	
Duration of surgery, h				0.63
≤2 h	1238 (74.0)	659 (73.1)	579 (75.1)	
2-3 h	235 (14.0)	132 (14.6)	103 (13.4)	
>3 h	200 (12.0)	111 (12.3)	89 (11.5)	
LOS, d	8 (5–14)	9 (6–14)	8 (5–13)	0.01

Data are median (quartile) unless otherwise specified.

SpO_2_: saturation of peripheral oxygen; ASA: American society of anesthesiologists; LOS: length of stay.

^*^Cohort 1 versus cohort 2; ^#^this category included general anesthesia alone and general anesthesia combined with regional blockade.

**Table 3:** Incidence of PPCs with LOS according to surgical specialties.

	All patients	PPCs, *n* (%)	LOS (day)		*P *
			With PPCs (*n* = 163)	Without PPCs (*n* = 1510)	
Total	1673	163 (9.7)	16 (10–23)	8 (5–13)	<0.001
Surgical specialty					
Orthopaedics	303	10 (3.3)	22 (14–31)	11 (6–17)	0.03
Breast	72	2 (2.8)	20 (19–21)	4 (3–8)	<0.001
Gynaecology	208	16 (7.7)	10 (7–19)	6 (4–8)	<0.001
Vascular	33	1 (3.0)	16 (16–16)	9 (7–13)	0.45
Upper gastrointestinal	47	19 (40.4)	18 (16–21)	17 (13–20)	0.68
Lower gastrointestinal	136	16 (11.8)	17 (15–28)	11 (6–15)	<0.001
Hepatobiliary	229	31 (13.5)	24 (16–33)	8 (5–14)	<0.001
Urology	168	7 (4.2)	16 (13–38)	8 (6–11)	0.16
Kidney	56	8 (14.3)	13 (10–17)	10 (7–14)	0.65
Head and neck	147	9 (6.1)	4 (3–7)	7 (5–9)	0.21
Thoracic	49	19 (38.8)	12 (10–15)	14 (9–16)	0.36
Neurosurgery	36	8 (22.2)	22 (12–27)	18 (12–25)	0.40
Endocrinology	147	14 (9.5)	8 (7–14)	7 (6–8)	0.11
Others	41	4 (2.4)	10 (7–26)	7 (5–9)	0.16

Data are median (quartile) unless otherwise specified.

PPCs: postoperative pulmonary complications; LOS: length of stay.

**Table 4:** Distribution of results of independent variables in cohort 1.

	Number of patients	Number (%) of patients with PPCs	*P *
Age, yr			<0.001
<59	670	65 (9.7)	
≥60	232	47 (20.3)	
ASA physical status			<0.001
1	464	20 (4.3)	
2	380	77 (20.3)	
3	54	13 (24.1)	
4	3	1 (33.3)	
5	1	1 (100.0)	
Smokers			0.001
No	753	79 (10.5)	
Yes	149	33 (22.1)	
Respiratory infection in the last month			<0.001
No	886	101 (11.4)	
Yes	16	11 (68.8)	
Diabetes			0.001
No	846	97 (11.5)	
Yes	56	15 (26.8)	
COPD			<0.001
No	890	106 (11.9)	
Yes	12	6 (50.0)	
Cirrhosis			0.014
No	895	109 (12.2)	
Yes	7	3 (42.9)	
Stroke/transient ischaemic attack			0.001
No	887	106 (12.0)	
Yes	15	6 (40.0)	
Preoperative antibiotic use			<0.001
No	298	19 (6.4)	
Yes	604	93 (15.4)	
Preoperative SpO_2_, %			<0.001
≥96	845	92 (10.9)	
<96	57	20 (35.1)	
Preoperative anemia			0.007
No	837	97 (11.6)	
Yes	65	15 (23.1)	
Preoperative LOS, d			0.002
<2	126	5 (4.0)	
≥2	776	107 (13.8)	
Surgery site			<0.001
Peripheral	620	36 (5.8)	
Abdominal	243	56 (23.0)	
Intrathoracic	39	20 (51.3)	
Anesthesia			<0.001
Regional	238	10 (4.2)	
General	664	102 (15.4)	
Intraoperative nasogastric tube			<0.001
No	847	85 (10.0)	
Yes	55	27 (49.1)	
Intraoperative bladder catheter			<0.001
No	466	28 (6.0)	
Yes	436	84 (19.3)	
Intraoperative central venous catheter			<0.001
No	821	85 (10.4)	
Yes	81	27 (33.3)	
Intraoperative blood loss, mL			<0.001
<100	636	32 (5.0)	
≥100	266	80 (30.1)	
Intraoperative blood transfusion			<0.001
No	875	102 (11.7)	
Yes	27	10 (37.0)	
Duration of surgery, h			<0.001
≤2 h	659	47 (7.1)	
>2 to 3 h	132	27 (20.5)	
>3 h	111	38 (34.2)	
Postoperative SpO_2_, %			<0.001
≥96	891	101 (11.3)	
<96	11	11 (100.0)	
Postoperative leucocyte, 10^9^/L			<0.001
<4	6	2 (33.3)	
4–10	665	47 (7.1)	
>10	231	63 (27.3)	
Postoperative anemia			0.009
No	793	90 (11.3)	
Yes	109	22 (20.2)	
Postoperative fasting blood glucose, mmol/L			<0.001
≤6.1	778	50 (6.4)	
>6.1	124	62 (50.0)	
Postoperative creatinine, *μ*mol/L			<0.001
≤115	872	99 (11.4)	
>115	30	13 (43.3)	
Postoperative albumin, g/L			<0.001
<35	244	85 (34.8)	
≥35	658	27 (4.1)	
Postoperative ventilation			<0.001
No	878	97 (11.0)	
Yes	24	15 (62.5)	
Postoperative vasoactive drug			<0.001
No	893	105 (11.8)	
Yes	9	7 (77.8)	

Smoking: defined as patients who smoked up to 1 year before surgery; Anemia: defined as hemoglobin <100 g/L.

PPCs: postoperative pulmonary complications; ASA: American society of anesthesiologists; COPD: chronic obstructive pulmonary disease; SpO_2_: saturation of peripheral oxygen; LOS: length of stay.

**Table 5:** Independent predictors of risk factors for PPCs.

	OR (95% CI) *n* = 902	*β* coefficient	Risk score
Respiratory infection in the last month	7.03 (1.66–29.80)	1.950	20
Smokers	2.37 (1.27–4.42)	0.861	9
Preoperative antibiotic use	0.238 (0.11–0.54)	−1.436	−14
Preoperative SpO_2_ <96%	5.56 (2.38–12.98)	1.715	17
Surgery site			
Peripheral	1		
Abdominal	2.88 (1.49–5.59)	1.058	11
Intrathoracic	12.20 (4.61–32.28)	2.501	25
Intraoperative blood loss ≥100 mL	3.00 (1.64–5.50)	1.100	11
Postoperative fasting blood glucose >6.1 mmol/L	2.60 (1.91–3.54)	0.956	10
Postoperative albumin <35 g/L	4.21 (2.24–7.92)	1.438	14
Postoperative ventilation	7.20 (1.96–26.45)	1.975	20

PPCs: postoperative pulmonary complications; SpO_2_: saturation of peripheral oxygen.

**Table 6:** Distribution of PPCs risk index scores in patients.

	Risk class			
	Low risk (<13 points)	Intermediate risk (13–30 points)	High risk (31–42 points)	Extremely high risk (>42 points)
Cohort 1, *n* (%)	672 (74.5)	127 (14.1)	67 (7.4)	36 (4.0)
Cohort 2, *n* (%)	577 (74.8)	110 (14.3)	54 (7.0)	30 (3.9)
Rate of PPCs in cohort 1, *n* (%)	21 (3.1)	22 (17.3)	38 (56.7)	31 (86.1)
Rate of PPCs in cohort 2, *n* (%)	5 (0.9)	17 (15.5)	12 (22.2)	17 (56.7)

PPCs: postoperative pulmonary complications.

**Table 7:** Interventions to reduce the risk of PPCs.

*Preoperative interventions *	
A careful history taking and physical examination	
Encourage cessation of smoking for at least 2 months	
Appropriate use of antibiotics and delay surgery if respiratory infection is present	
Recommend a regular exercise program (e.g., walking, upper limb exercises, swimming, pool exercises, etc.)	
Treat patients with established asthma with inhaled corticosteroids	
Treat patients with established COPD with regular bronchodilators	

*Intraoperative interventions *	
Substitute less ambitious procedure for upper abdominal or thoracic surgery when possible	
Minimize blood loss	
Limit duration of surgery to less than 3 hr	
Whenever possible, use spinal or epidural anesthesia	

*Postoperative interventions *	
Recommend regular lung expansion modalities such as deep breathing exercises	
Perform selective decompression of abdominal contents using nasogastric tube if patient is experiencing symptomatic gastric distension	
As soon as possible after surgery, have the patient sit up in a chair	

PPCs: postoperative pulmonary complications; COPD: chronic obstructive pulmonary disease.
